# Synergistic effects of SAE/CaCO_3_ on enhancing sulfate resistance in High-Strength cementitious composites

**DOI:** 10.1038/s41598-025-17550-9

**Published:** 2025-10-02

**Authors:** Lei Ren, Puyan Wang, Siyi Fang, Weijun Zhong, Jianwei Ren, Jian Zhou, Mingfang Ba

**Affiliations:** 1 Ningbo Electric Power Design Institute Co., Ltd, Ningbo, China; 2 China Academy of Building Research Co., Ltd., Ningbo, China; 3 School of Civil & Environmental Engineering and Geography Science, Ningbo University, Ningbo, China

**Keywords:** Styrene acrylic emulsion, CaCO_3_, High-strength cementitious composites, Sulfate attack resistance, Microscopic mechanism, Engineering, Materials science

## Abstract

The composite incorporation of polymer and calcium carbonate (CaCO_3_) in cementitious matrices demonstrates effective toughening effects on high-strength cementitious composites (HSCC). However, its resistance to salt ion erosion remains unverified, which is critical for construction materials in coastal environments and underground engineering. This study systematically evaluates the sulfate attack resistance of SAE/CaCO_3_ modified HSCC through mass variation analysis, ultrasonic monitoring, durability coefficient determination, and surface morphology characterization. Advanced microstructural investigations including MIP, XRD, DSC, and SEM were employed to elucidate the synergistic mechanisms of SAE/CaCO_3_ in enhancing durability of the cement matrix. Results reveal that SAE/CaCO_3_ modified HSCC exhibits exceptional sulfate resistance. Specifically, under sulfate attack, high-SAE dosage specimens showed lower mass fluctuation and less mechanical property degradation compared to control groups. The primary mechanism stems from SAE-induced pore structure refinement. This study elucidates critical structure-performance relationships in SAE/CaCO_3_ modified HSCC systems, offering dual benefits of special engineering environment durability enhancement and mechanistic insights for polymer-cement composite optimization.

## Introduction

Advancements in modern concrete technology and the rapid exploitation of marine resources and subsurface environments have intensified demands for high-strength concretes tailored to extreme engineering conditions^1,2^. However, infrastructure development in these complex environments, particularly marine settings, faces critical challenges. Concrete structures in these engineering settings endure persistent physical erosion from water molecule penetration and inorganic salt ion migration induced by temperature/humidity variations, coupled with chemical deterioration from salt corrosion^3^. Studies have revealed that synergistic physical-chemical interactions accelerate structural performance deterioration^4^. Consequently, concretes for extreme environments must demonstrate superior ductility and resistance to harmful ion penetration. These requirements highlight the necessity to enhance both toughness and durability in high-strength cement matrices for practical engineering applications.

Significant research efforts have been directed towards enhancing the sulfate attack resistance of cementitious materials. Du et al. demonstrated that incorporating NaCl can markedly improve the sulfate resistance of concrete^5^. This improvement is attributed to NaCl’s dual function: inhibiting SO_4_^2−^ ingress and consuming corrodible cement hydration products, thereby reducing the formation of deleterious corrosion products within the concrete. However, the addition of NaCl was found to reduce the compressive strength of concrete and mitigate the development of the damage layer thickness. Separately, Ogawa et al. investigated the influence of limestone and calcium sulfate content on sulfate resistance of ground granulated blast furnace slag (GGBS) blended cement^6^. Their findings revealed that limestone incorporation and increased calcium sulfate content facilitate the formation of monocarboaluminate and ettringite phases prior to sulfate solution immersion. This pre-formation effectively inhibits further ingress of external sulfate ions during subsequent exposure. It is evident that current strategies for improving sulfate resistance in cementitious systems primarily focus on inorganic salt modifications. While this approach effectively enhances resistance to sulfate ion penetration, its effectiveness in enhancing the toughness of the cementitious matrix is limited.

The hybridization of organic and inorganic phases, which combines their respective advantages, offers a novel approach to tailoring the engineering properties of composite cementitious materials^7,8^. The polymer industry’s evolution has facilitated the direct utilization of polymeric additives in concrete production^8^. Responding to modern concrete technology demands, Polymer Modified Cement Composites (PMCC) have emerged as advanced materials. Commonly employed polymers include Styrene Butadiene Rubber (SBR), Styrene Acrylate Emulsion (SAE), Epoxy Resin (ERE), and Ethylene Vinyl Acetate (EVA)^9,10^. These polymer modifications influence cementitious materials through multiple mechanisms: regulating cement hydration^11^optimizing pore structure^12^and modifying the morphology of hydration products^13^thereby changing workability, mechanical performance, and ductility^14,15^. Although different polymers exhibit varied interaction mechanisms, their effectiveness in improving cementitious material flexibility and toughness remains well-documented^16,17^. Notably, SAE demonstrates particular promise due to its dual benefits in enhancing material fluidity/ductility and cost-effectiveness for practical engineering applications. To further improve impermeability, CaCO_3_ has been incorporated into SAE-modified cement matrices^18^. Typically added at 5% of binder mass^19^CaCO_3_ functions as both inert filler and reactive additive. Research has confirmed its capability to refine pore structures, enhance impermeability/mechanical strength, and accelerate early-age strength development^19,20^.

Although the enhanced performance of SAE/CaCO_3_ modified High-Strength Cementitious Composites (HSCC) against deleterious ion ingress is attracting considerable attention, the underlying mechanisms remain unclear and warrant further investigation. Addressing the erosion challenges in special engineering environments, this study evaluates sulfate attack resistance in SAE/CaCO_3_ modified HSCC through comprehensive metrics: mass variation, ultrasonic parameters, corrosion/penetration coefficients, and morphological characteristics. Advanced microstructural analyses including MIP, XRD, DSC, and SEM elucidate the synergistic mechanisms of SAE and CaCO_3_ in enhancing erosion resistance. This investigation establishes structure-property correlations for SAE/CaCO_3_ modified HSCC, providing both technical guidance for special engineering environment applications and theoretical foundations for optimizing polymer-modified cementitious materials.

## Materials and methods

### Materials and mix proportions

**Fig. 1:**
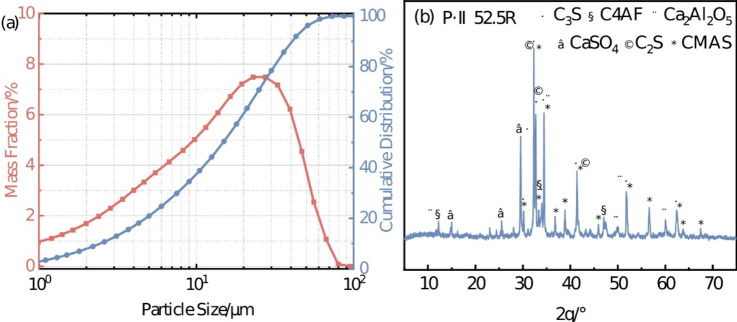
Particle size distribution (**a**) and XRD pattern (**b**) of Type P·II 52.5R Portland cement.

Portland cement (Type P·II 52.5R, Conch Brand) was utilized, with its chemical composition and loss on ignition (LOI) values listed in Table 1. Figure 1 illustrates the laser particle size distribution curve and XRD pattern of the cement, indicating a typical particle size range of 1–100 μm with peak distribution around 10–20 μm (a), and revealing dominant crystalline phases including alite (C_3_S), belite (C_2_S), tricalcium aluminate (C_3_A), and ferrite (C4AF) within the CaO-MgO-Al_2_O_3_-SiO_2_ (CMAS) system (b). Styrene-acrylate emulsion (SAE, BASF’s Agility PS608) was employed, synthesized via emulsion copolymerization of styrene and acrylate monomers, presenting as a milky liquid with bluish opalescence. Nanoscale CaCO_3_ and defoamer were supplied by McLean Chemical Co., Ltd. The CaCO_3_ exhibited an average particle size of 50 nm, while the defoamer was an organic silicon-based defoamer (OSD). Key performance parameters of SAE and OSD are detailed in Table 2.

**Table 1:** The chemical compositions of type P·II 52.5R Portland cement (wt%).

CaO	SiO_2_	Al_2_O_3_	SO_3_	Fe_2_O_3_	MgO	K_2_O	LOI
62.621	21.229	5.418	3.696	3.599	1.552	0.939	1.93

**Table 2:** Performance parameters of SAE and OSD.

No.	Viscosity/ mPa s	pH	Solid content/ %	Density/g/cm^3^
SAE	500–2000 (23 °C)	6.5 –8.5	49–51	1.04
OSD	160–200	7.0–8.0	11–13	–

**Table 3:** The mixture composition of the HSCC (kg/m^3^).

No.	Cement	CaCO_3_	Water	SAE	OSD
PC	1525.9	80.3	481.9	0	3.21
SAE2	1525.9	80.3	449.8	64.25	3.21
SAE3	1525.9	80.3	433.7	96.35	3.21
SAE4	1525.9	80.3	417.65	128.5	3.21
SAE5	1525.9	80.3	401.6	160.6	3.21

Specimens with a water-to-cement ratio (W/C) of 0.3 were prepared, where the polymer-to-cement (P/C) ratios were set at 0%, 2%, 3%, 4%, and 5%, designated as PC, SAE2, SAE3, SAE4, and SAE5, respectively. The corresponding mix proportions of SAE/CaCO_3_ modified HSCC are detailed in Table 3.

### Sample preparation

**Fig. 2:**
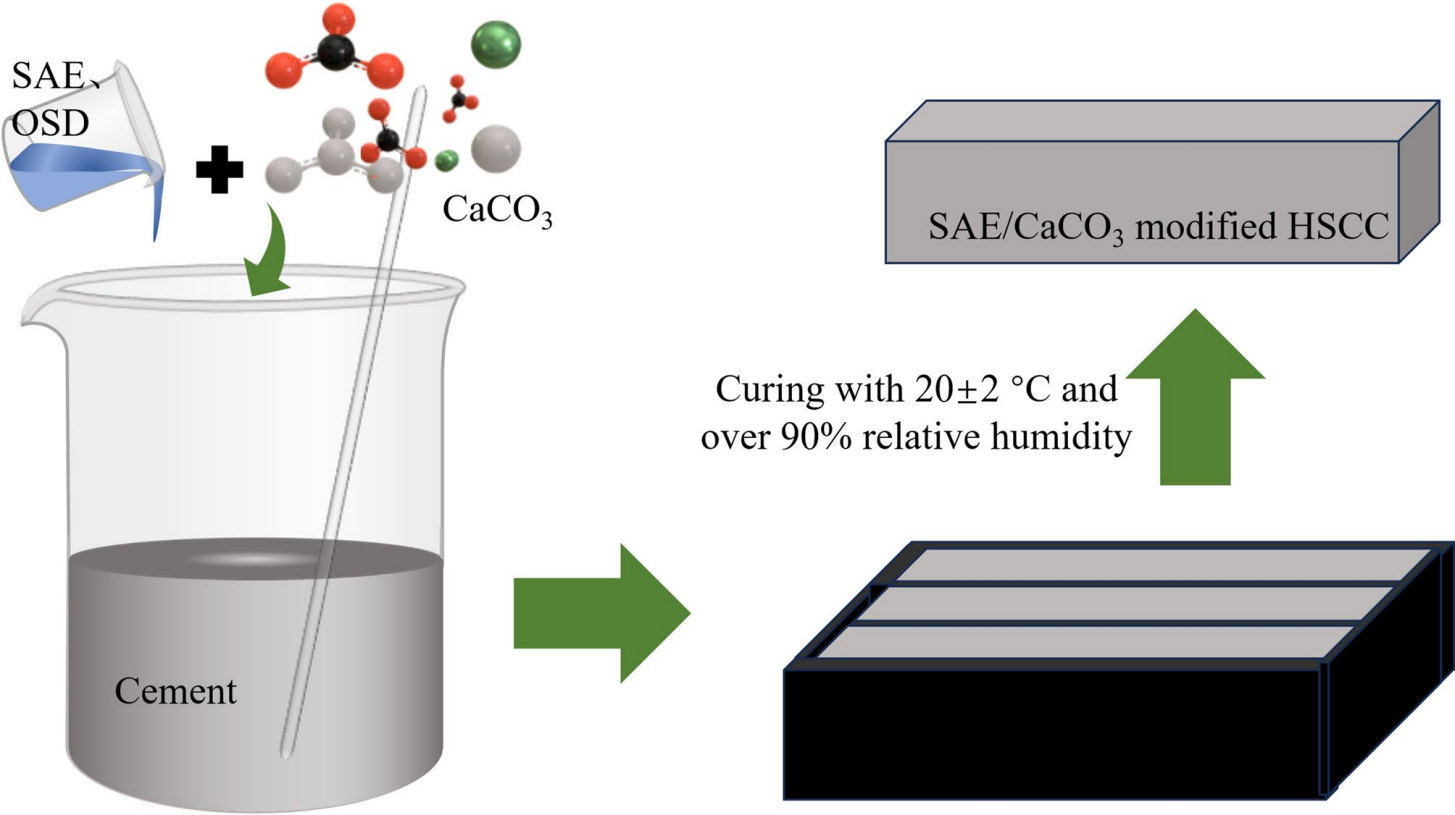
Schematic diagram of the preparation of SAE/CaCO_3_ modified HSCC.

The Portland cement was initially blended with 5% nanoscale CaCO_3_ (by total binder mass). Following the mix proportions outlined in Table 3, SAE, OSD, and water were subsequently incorporated through standard mixing procedures as schematically illustrated in Fig. 2. The fresh cement paste was cast into prismatic molds (40 × 40 × 160 mm^3^. After 24-h ambient curing at room temperature, specimens were demolded and transferred to a controlled curing chamber maintaining standard conditions (20 ± 2 °C temperature, > 90% relative humidity) until the 27th day. The cured specimens were then subjected to sulfate attack resistance testing.

### Testing methods

#### Sulfate attack resistance testing

**Fig. 3:**
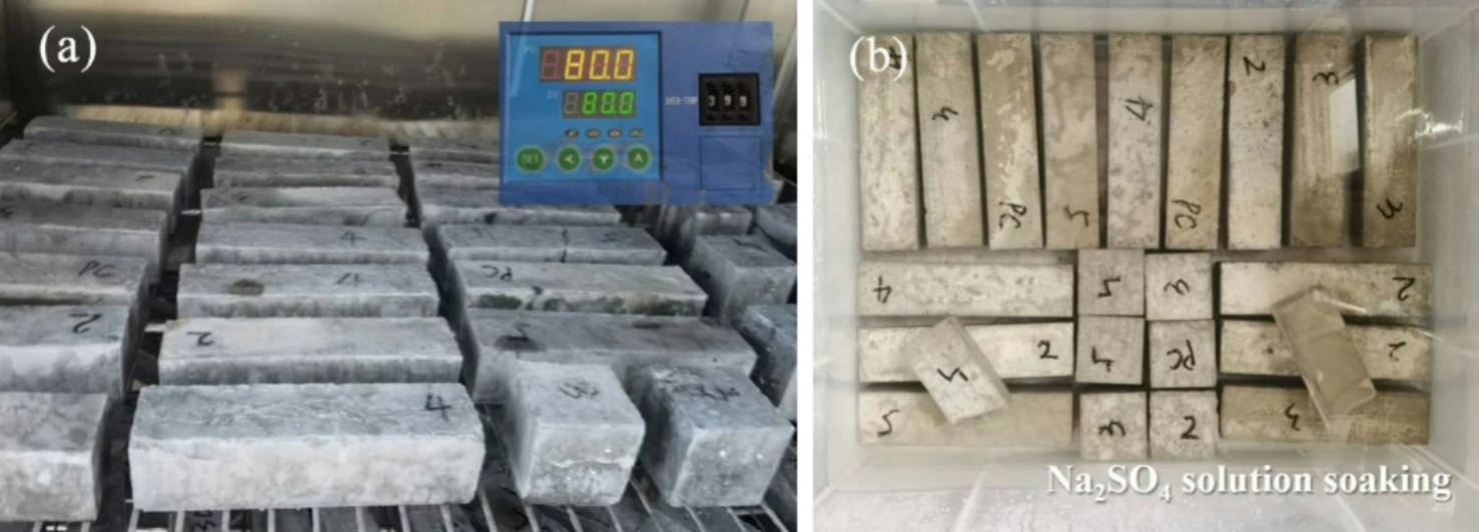
Diagram of sulfate wet-dry cycle test: (**a**) drying of specimens; (**b**) immersion of specimens.

The sulfate resistance of specimens was evaluated through wet-dry cycle test as stipulated in *Standard for Test Methods of Long-Term Performance and Durability of Ordinary Concrete* (GB/T 50082 − 2009)^21^. Specimens designated for cycling were extracted at 28 days of curing, surface-dried, and placed in an oven at 80 ± 5 °C for 48 h. After drying, specimens were cooled to ambient temperature in a desiccated environment. A 5% mass concentration Na_2_SO_4_ solution (sufficient to submerge specimens by 20 mm depth) was prepared. The immersion phase lasted 15 ± 0.5 h, after which specimens were air-dried for 1 h and immediately re-dried at 80 ± 5 °C for 6 h. Post-drying cooling to room temperature (2 h duration) completed each cycle. Each full cycle spanned 24 ± 2 h, with cumulative cycles conducted for 15 times (C15 group) and 30 times (C30 group), as diagrammed in Fig. 3. Control groups (B15/B30) remained under standard curing until comparative strength testing. The naming rules for different control groups during the experiment are shown in Table 4. And the durability coefficient was calculated per Eq. 1.

1 $${K_f} = \frac{{{f_{cn}}}}{{{f_{c0}}}} \times 100$$

where *K*_*f*_ is the strength corrosion resistance coefficient (%), *f*_*cn*_ is the strength measurement value (MPa) of the cement sample after N wet-dry cycles under sulfate corrosion, and *f*_*c0*_ is the strength measurement value (MPa) of a set of standard curing control specimens of the same age as the sulfate-corroded specimens.

**Table 4:** The definition of test group.

Designation	Curing/exposure condition	Description
B15	Standard curing (Baseline)	Specimens were cured normally until the test group completed 15 cycles of sulfate wet-dry attack
B30	Standard curing (Baseline)	Specimens were cured normally until the test group completed 30 cycles of sulfate wet-dry attack
C15	Sulfate Wet-Dry Cycling	Specimens were subjected to 15 cycles of sulfate wet-dry attack
C30	Sulfate Wet-Dry Cycling	Specimens were subjected to 30 cycles of sulfate wet-dry attack

**Fig. 4:**
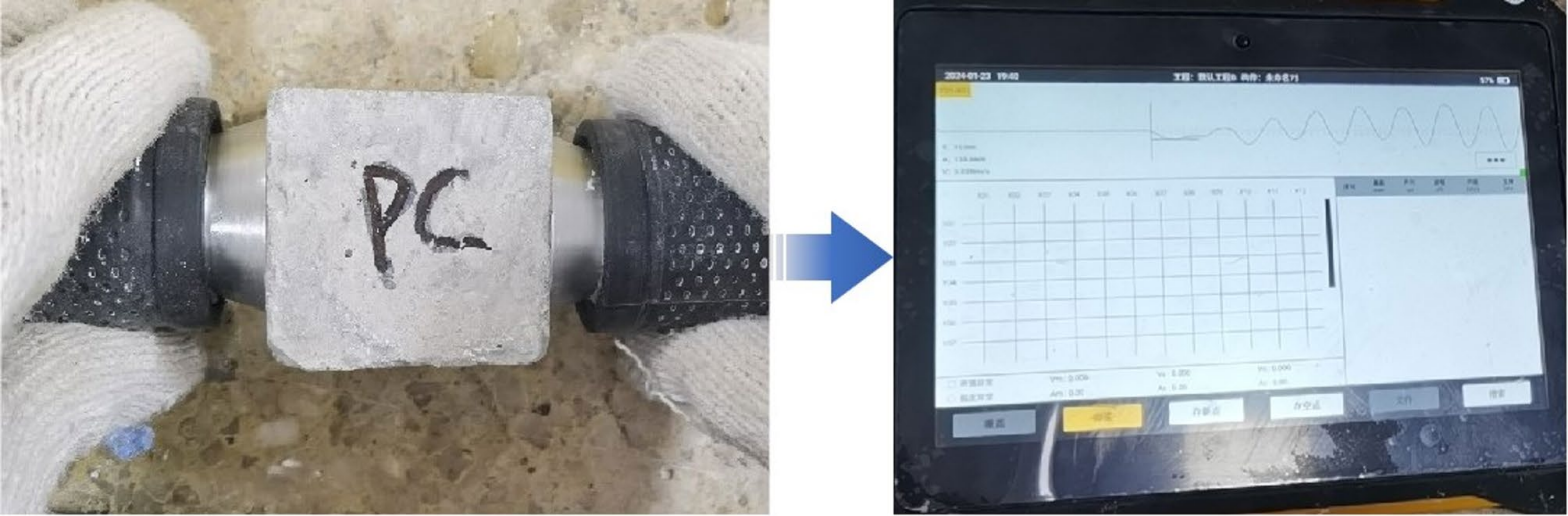
Ultrasonic testing instrument test chart.

Mass measurements and ultrasonic wave velocity tests (HC-F900 concrete ultrasonic tester, Beijing Haichuang Hi-Tech Co.) were performed after each cycle (Fig. 4). Mass variation rate (Eq. 2) and internal damage index (Eq. 3) were quantified to assess degradation progression.

2 $${w_n}=\frac{{{m_n} - {m_0}}}{{{m_0}}} \times 100$$

3 $${V_R}=\frac{{{V_n}}}{{{V_0}}}$$

where *w*_*n*_ is the mass change rate of cement samples (%), *m*_*n*_ is the mass (g) of specimens after n cycles of wet-dry alternation under sulfate attack, *m*_*0*_ is the mass (g) of specimens before any wet-dry cycles, *V*_*R*_ is the relative wave velocity, *V*_*n*_ is the ultrasonic wave velocity (km/s) of specimens after n cycles of wet-dry alternation, and *V*_*0*_ is the ultrasonic wave velocity (km/s) of specimens before erosion.

#### Mechanical property testing

1. Mechanical strength test

The flexural and compressive strengths of the test specimens from Group B15, C15, B30, and C30 in Section “Sulfate attack resistance testing” were measured using a WAW-600 C microcomputer-controlled electro-hydraulic servo universal testing machine. The test standard refers to *Test Method for Compressive and Flexural Strength of Cement Mortar (ISO Method)* (GB/T 17671−2021)^22^. A three-point bending fixture with a 100 mm span was installed in the machine to obtain load-displacement curves and determine flexural strength. The 40 × 40 × 160 mm^3^ specimens were loaded in three-point bending configuration under force-controlled mode, with the force accumulating at 60 N/s until the load decreased by 80% of its maximum value. The maximum load was recorded to calculate the flexural strength. Following the three-point bending tests, compression tests were conducted on the two fractured prismatic segments using the same machine. The force was applied in force-controlled mode at a rate of 2400 N/s until the load declined by 50%. Each group underwent three flexural tests and six compressive tests^22^.

(2) Matrix toughness analysis

The variation in matrix toughness was characterized through changes in the flexural-to-compressive strength (F/C) ratio and fracture energy. The F/C ratio is defined as the quotient between the flexural strength and compressive strength obtained from Section “Mechanical property testing”(1). Fracture energy calculation was directly related to the evolution of stress-strain curves during the entire flexural testing process. The study employed Digital Image Correlation (DIC) non-contact strain-displacement measurement technology to capture displacement variations at the center of the specimen’s bottom surface throughout the stress-strain process until complete fracture failure. The fracture energy evolution of SAE/CaCO_3_ modified HSCC was calculated using Formula 4 based on the experimentally obtained load-displacement curves^23^.

4 $${G_f}\frac{{\int_{0}^{{{\delta _0}}} {P\left( \delta \right){\text{d}}\left( \delta \right)+mg{\delta _0}} }}{{{A_{lig}}}}=\frac{{{W_0}+mg{\delta _0}}}{{hb}}$$

where *δ*_*0*_ is the deflection at the initial crack point of the specimen, *m* is the mass of the specimen, *A*_*lig*_ is the area of the fracture section of the specimen, *h* and *b* are the height and width of the cross-section of the specimen, and *W*_*0*_ is the initial crack energy.

#### Microscopic mechanism testing

Specimens subjected to 15 times and 30 times sulfate wet-dry cycles underwent hydration termination via isopropanol immersion. Subsequent drying was conducted at 30 °C for ≥ 30 min. Fresh fracture surfaces were selected for SEM analysis, while intact blocks were reserved for MIP testing. Remaining material was pulverized to < 21 μm particle size using an agate mortar for XRD and DSC characterization of corrosion products.

**Fig. 5:**
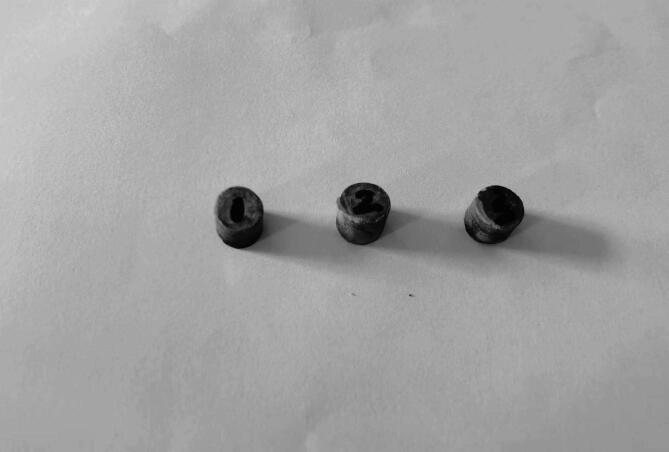
Cylindrical block specimens used in MIP tests.

For pre-erosion pore structure analysis, cylindrical samples (< 1 cm^2^ volume, < 1 cm base diameter) were extracted from SAE/CaCO_3_ modified HSCC (Fig. 5).

SEM analysis: Pre-dried specimens were coated with gold films via plasma spraying and examined using a COXEM EM-30AX Plus scanning electron microscope (South Korea) operated at 12 keV accelerating voltage.

XRD analysis: Phase identification was conducted using a Bruker D2 Phaser X-ray diffractometer with Cu-Kα radiation (λ = 0.15419 nm). Scans were performed over 10°–0° 2θ range with 0.02° step size at ambient conditions.

DSC Analysis: Thermal behavior was evaluated via NETZSCH DSC 214 differential scanning calorimeter under nitrogen atmosphere (Testing temperature: 30–400 °C, 20 °C/min heating rate).

MIP analysis: Pore structure quantification was implemented through Micromeritics AutoPore V 9620 mercury intrusion porosimeter with maximum intrusion pressure of 61,000 psi. The Washburn equation was applied with mercury surface tension (0.485 N/m) and contact angle (130°), yielding measurable pore diameters ≥ 3 nm^8^. No surface modification or consistency treatments were performed.

## Results and discussions

### The regularity of mass variation

**Fig. 6:**
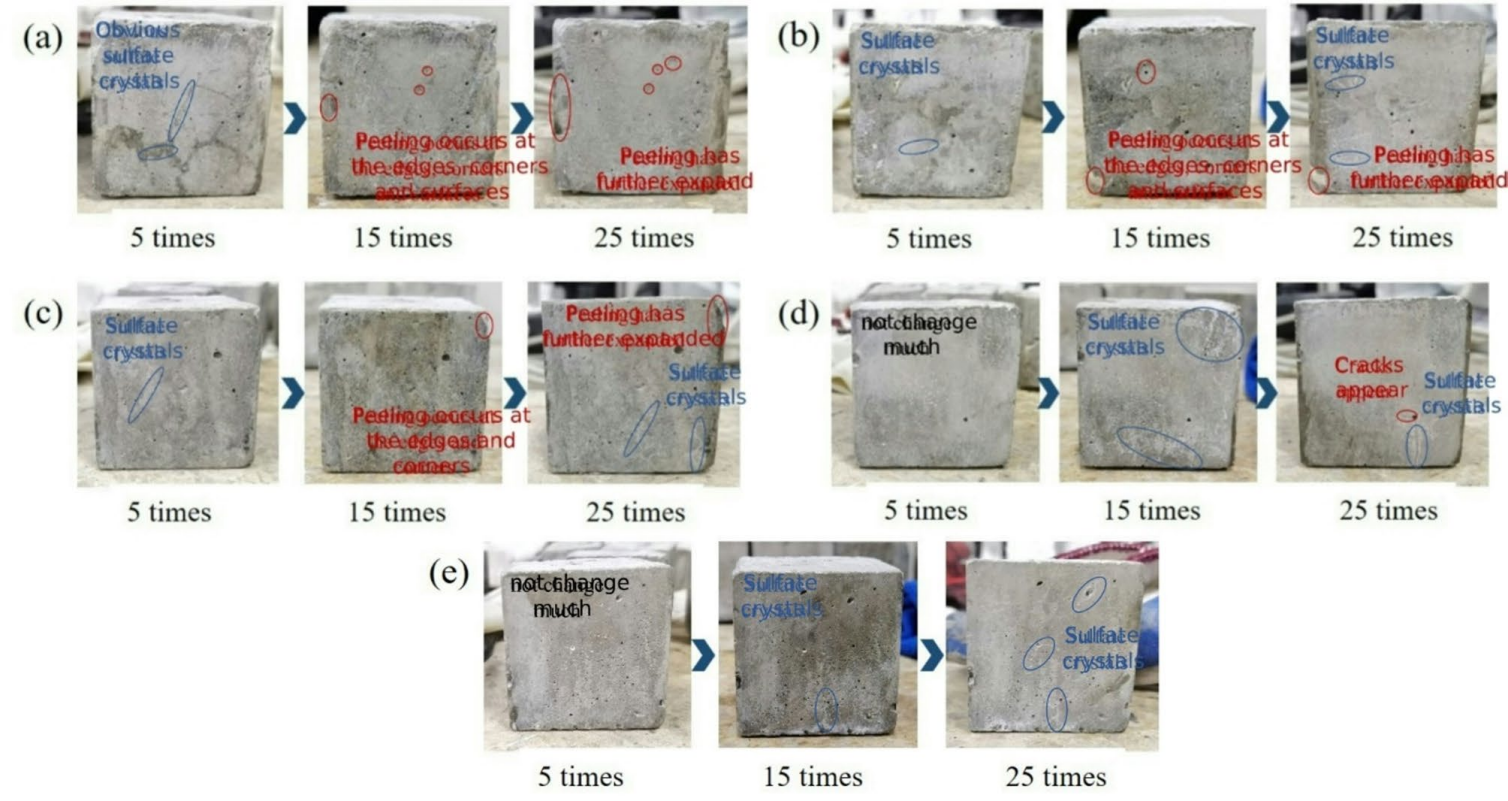
The apparent morphology of SAE/CaCO_3_ modified HSCC at different sulfate attack stages: (**a**) PC; (**b**) SAE2; (**c**) SAE3; (**d**) SAE4; (**e**) SAE5.

To visually assess the macroscopic damage evolution of SAE/CaCO_3_ modified HSCC under sulfate attack, the apparent morphology of specimens at various erosion stages was documented, with mass variation rate adopted as a key evaluation metric. Figure 6 illustrates the surface morphological evolution across erosion cycles. As observed in Fig. 6, distinct sulfate crystal precipitates formed on specimen surfaces, accompanied by localized spalling that intensified with cycling frequency. Notably, the unmodified PC specimens exhibited more pronounced delamination compared to SAE-modified samples.

**Fig. 7:**
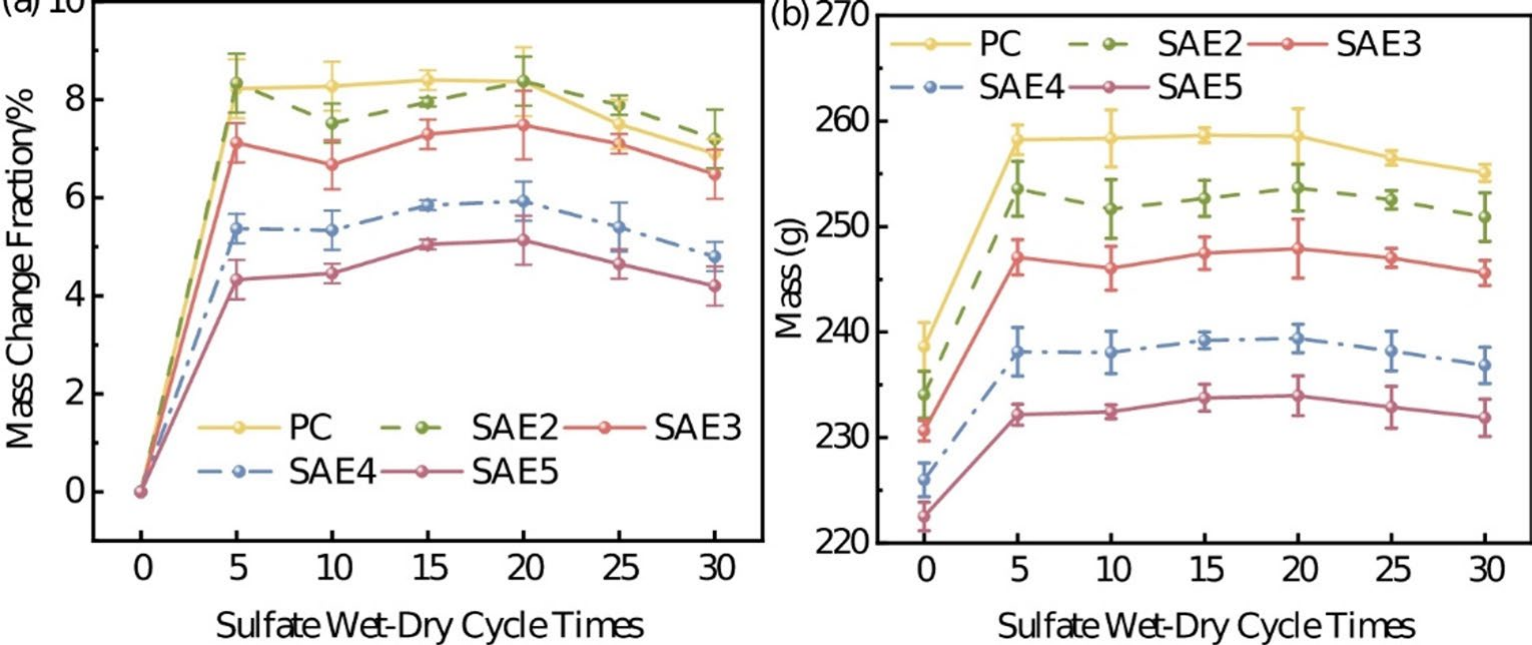
Mass change rate of SAE/CaCO_3_ modified HSCC at different stages of sulfate attack: (**a**) mass change fraction; (**b**) mass change.

Figure 7 presents the actual mass change and its rate for specimens subjected to 30 sulfate wet-dry cycles. All specimens demonstrated significant mass increase during the initial 5 cycles. Subsequently, a relative mass decrease was observed after approximately 10 cycles, followed by a recovery of the increasing trend, culminating in a final decrease. Nevertheless, the mass after 30 cycles remained higher than the initial value. The mass change rate of cement samples across different erosion stages reflects, to some extent, the deterioration mechanism underlying sulfate attack damage.

In the sulfate environment, the mass variation of concrete specimens can be attributed to two primary processes: a mass accumulation phase and a mass loss phase. The mass accumulation phase is characterized primarily by sulfate ingress and subsequent crystallization within capillary pores, initially densifying the matrix and thereby increasing specimen mass. Concurrent sulfate-hydrate reactions, such as ettringite (AFt) formation, further contribute to mass gain^24,25^. The mass loss phase manifests as microcracking and surface spalling induced by the expansion of corrosion products^26^. Particularly in later cycles, decomposition of CH and C-S-H phases occurs.

Analysis of Fig. 7 reveals that sulfate ingress and reaction product formation predominantly govern early-stage mass increase in SAE/CaCO_3_ modified HSCC. After 5 cycles, cracks and damage began to develop internally within the specimens, corresponding to changes in apparent morphology (Fig. 6). Continued mass increase beyond 10 cycles suggests persistent sulfate ingress, with the expansive corrosion products filling pores within newly formed cracks. When the accumulation of corrosion products reaches the internal stress limit of the cementitious matrix, cracks propagate further, leading to intensified internal structural damage within the specimens, consistent with findings reported in the literature^26,27^. It is evident that during sulfate attack, the mass accumulation and loss experienced by the cementitious matrix constitute a dynamic cyclic process involving alternating phases, rather than a singular mechanism.

Comparative analysis across varying SAE dosages (Fig. 7) demonstrates reduced mass fluctuation amplitudes with increased polymer content. Specifically, SAE5 exhibited a 39.13% lower rate of mass variation than PC after 30 cycles. This attenuation correlates with SAE-induced pore structure refinement. For PC specimens, higher initial porosity facilitates rapid sulfate penetration and extensive reaction product formation, amplifying mass gain. However, in SAE-modified systems, polymer gel networks obstruct capillary pore connectivity, reducing sulfate ingress and limiting expansive product generation. The synergistic CaCO_3_-SAE interaction further enhances pore blocking efficiency, decreasing mass variation rates proportionally to SAE dosage^27^. These findings confirm SAE’s critical role in mitigating sulfate attack through microstructural densification^28^.

### The changing regularity of flexural and compressive strength

**Fig. 8:**
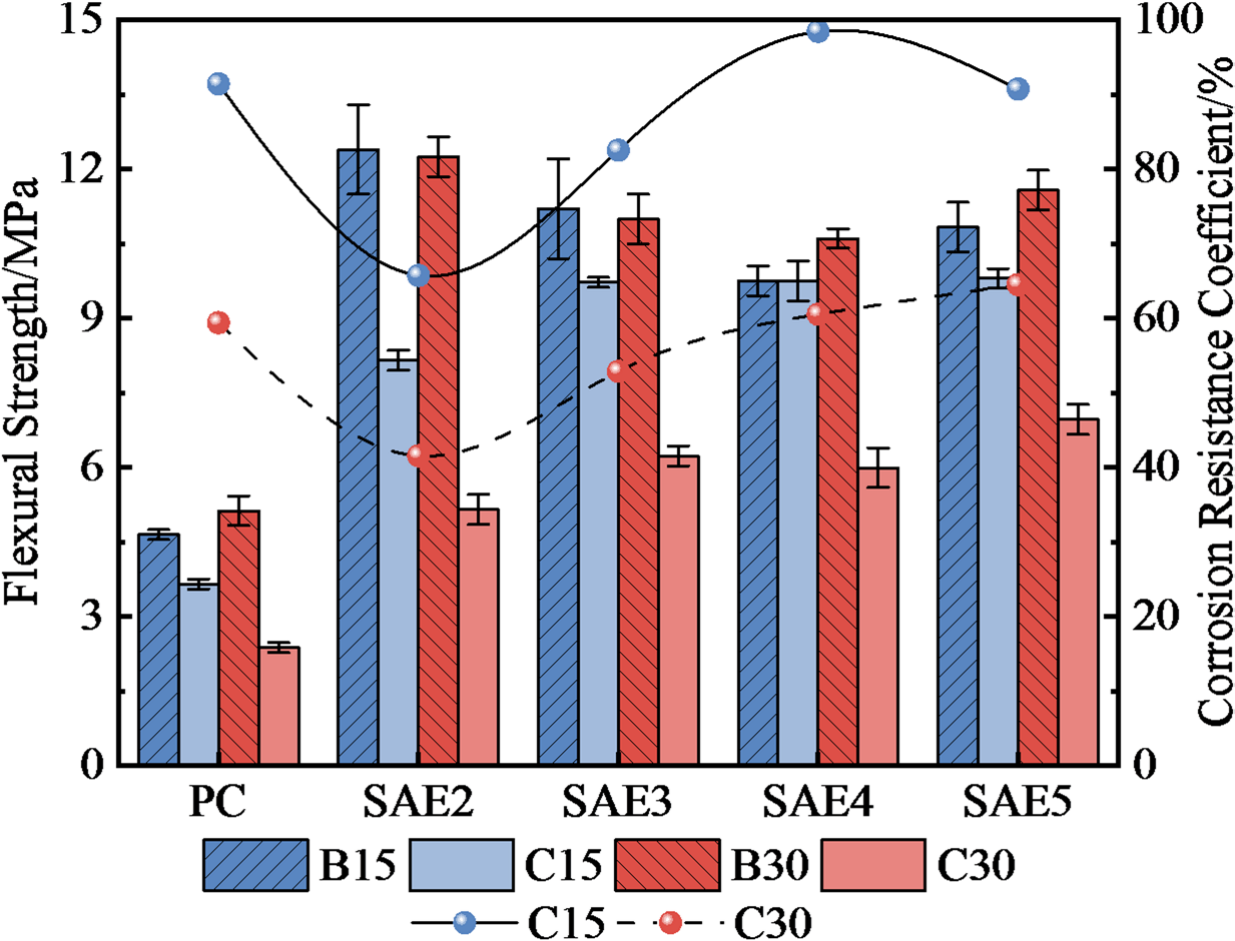
The flexural strength variation and corrosion resistance coefficient of SAE/CaCO_3_ modified HSCC at different sulfate erosion stages.

Figure 8 presents the flexural strength and corrosion resistance coefficients of SAE/CaCO_3_ modified HSCC after 15/30 sulfate wet-dry cycles. All specimens exhibited reduced flexural strength after sulfate erosion. PC specimens showed a smaller percentage decline (e.g., 40% after 30 cycles) because their initial strength was significantly lower (about 60% less than SAE groups), leaving limited room for further loss. Nevertheless, SAE-modified systems maintained higher absolute residual strength and superior durability coefficients (when SAE content was more than 4%), confirming that SAE enhances initial strength and sulfate resistance by densifying the microstructure.

**Fig. 9:**
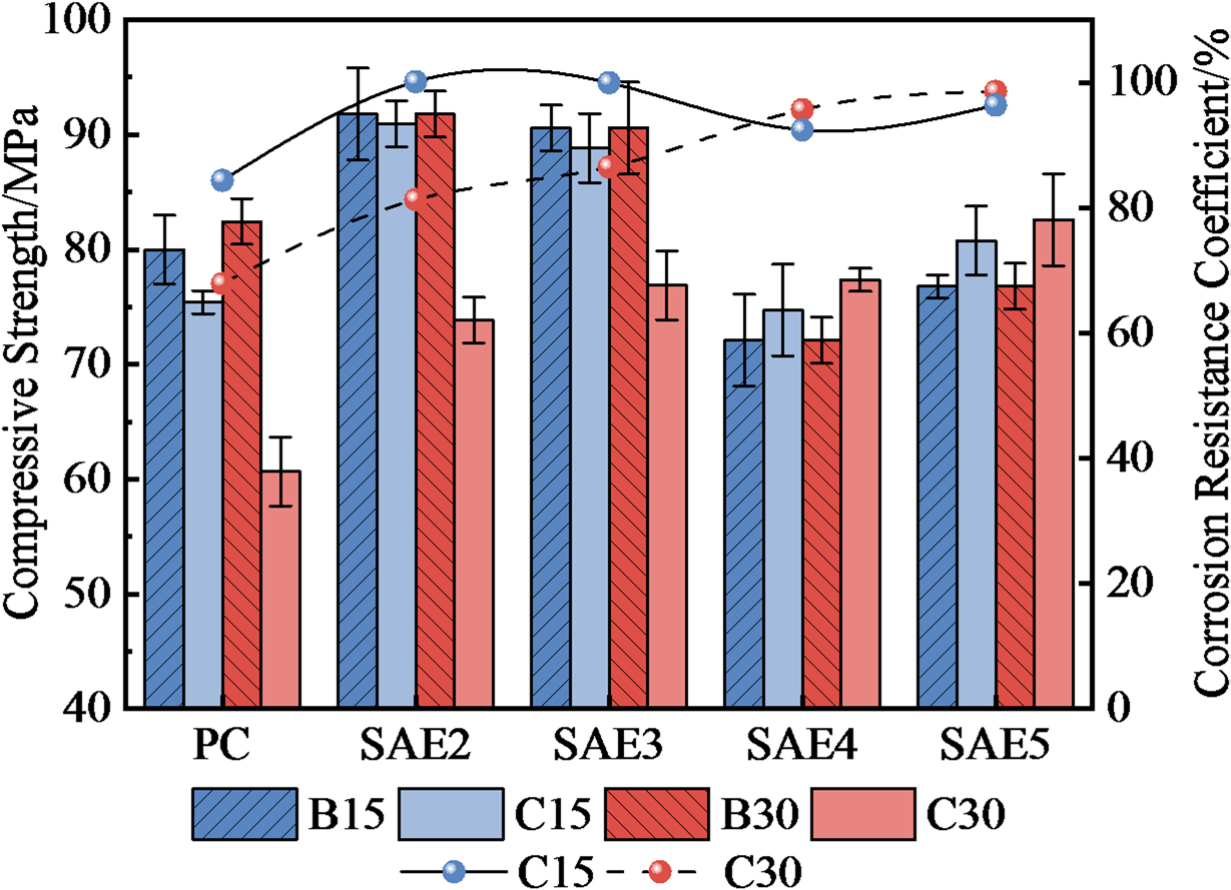
The compressive strength variation and corrosion resistance coefficient of SAE/CaCO_3_ modified HSCC at different sulfate erosion stages.

Figure 9 details the compressive strength evolution, where the compressive strength of SAE/CaCO_3_ modified HSCC more effectively elucidates the superior corrosion resistance of SAE within the cement matrix compared to flexural behavior. Specimens with ≥ 4% SAE content exhibited compressive strength increases post-cycling: SAE4 and SAE5 achieved 77.36 MPa (+ 7.3%) and 82.6 MPa (+ 7.55%), respectively, versus non-eroded counterparts. This strength gain correlates with SAE/CaCO_3_-induced pore refinement^27^. Sulfate attack induces the formation of varying amounts of corrosion products (e.g., AFt) within specimens. While these products densify the cement matrix, their formation simultaneously promotes microcracking and brittle failure. The incorporation of SAE mitigates sulfate ingress, thereby reducing AFt generation^29^. Concurrently, its polymeric encapsulation provides ductility that alleviates stress concentration arising from AFt-filled pores, namely, suppressing microcrack initiation. This mechanism explains the observed compressive strength increase in specimens SAE4 and SAE5. Collectively, these results demonstrate the superior sulfate corrosion resistance of the modified high-performance concrete.

### The variational law of toughness characteristics

Table 4 summarizes the flexural-to-compressive strength (F/C) ratios of SAE/CaCO_3_ modified HSCC after 15/30 sulfate wet-dry cycles. SAE-modified specimens consistently exhibited higher F/C ratios than PC, with the ratio increasing proportionally to SAE dosage. For instance, SAE5 demonstrated a 143.1% and 143.55% F/C ratio increase over PC in B15 (43-day curing) and B30 (58-day curing) control groups, respectively. Post-30-cycle exposure, SAE5 retained a 115.4% higher F/C ratio than PC, though the enhancement magnitude decreased by 34.1% points compared to early cycles, indicating prolonged sulfate exposure detrimentally impacts matrix ductility. This conclusion can likewise be derived from the variation of the fracture energy of the samples.

**Table 4:** The variation of the F/C strength ratios of SAE/CaCO_3_ modified HSCC at different sulfate erosion stages.

No.	PC	SAE2	SAE3	SAE4	SAE5
B15	0.058	0.135	0.124	0.135	0.141
C15	0.0485	0.090	0.1095	0.130	0.121
B30	0.062	0.133	0.121	0.147	0.151
C30	0.039	0.070	0.081	0.0775	0.084

**Fig. 10:**
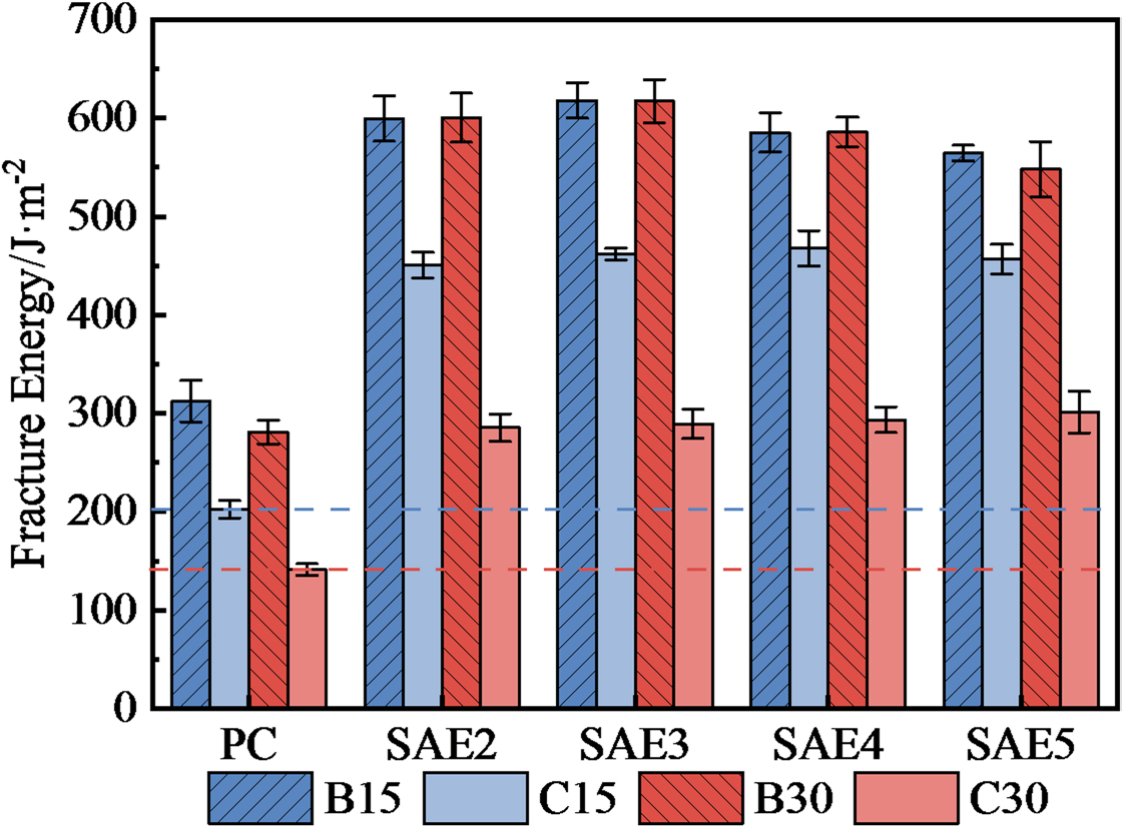
The variation of the fracture energy of SAE/CaCO_3_ modified HSCC at different stages of sulfate erosion.

Figure 10 details the fracture energy degradation, where all specimens exhibited significant toughness loss after 30 cycles: PC (– 49.7%), SAE2 (– 52.4%), SAE3 (– 53.1%), SAE4 (– 50.0%), and SAE5 (– 45.0%). And the SAE-induced fracture energy superiority over PC diminished with cycling severity. Specifically, after 15 sulfate cycles, SAE2 demonstrated a 122.5% higher fracture energy than PC, which reduced to 102.1% after 30 cycles. This declining trend was consistently observed across other samples: SAE3 (from 128.1 to 104.8%), SAE4 (from 131.0 to 107.8%), and SAE5 (from 125.6 to 113.3%), indicating progressive deterioration of polymer-cement interfacial bonding with extended sulfate exposure. This is because the corrosion products of sulfate are AFt and CaSO_4_. The generation of the corrosion products not only gives rise to micro-cracks within the cement matrix due to stress concentration, but also undermines the double-network gel structure formed by SAE and C–S–H gel, attenuating the stress diffusion effect of SAE as a flexible gel, and thus reducing the toughness characteristics of the cement matrix.

### The regularity of variation in structural homogeneity

**Fig. 11:**
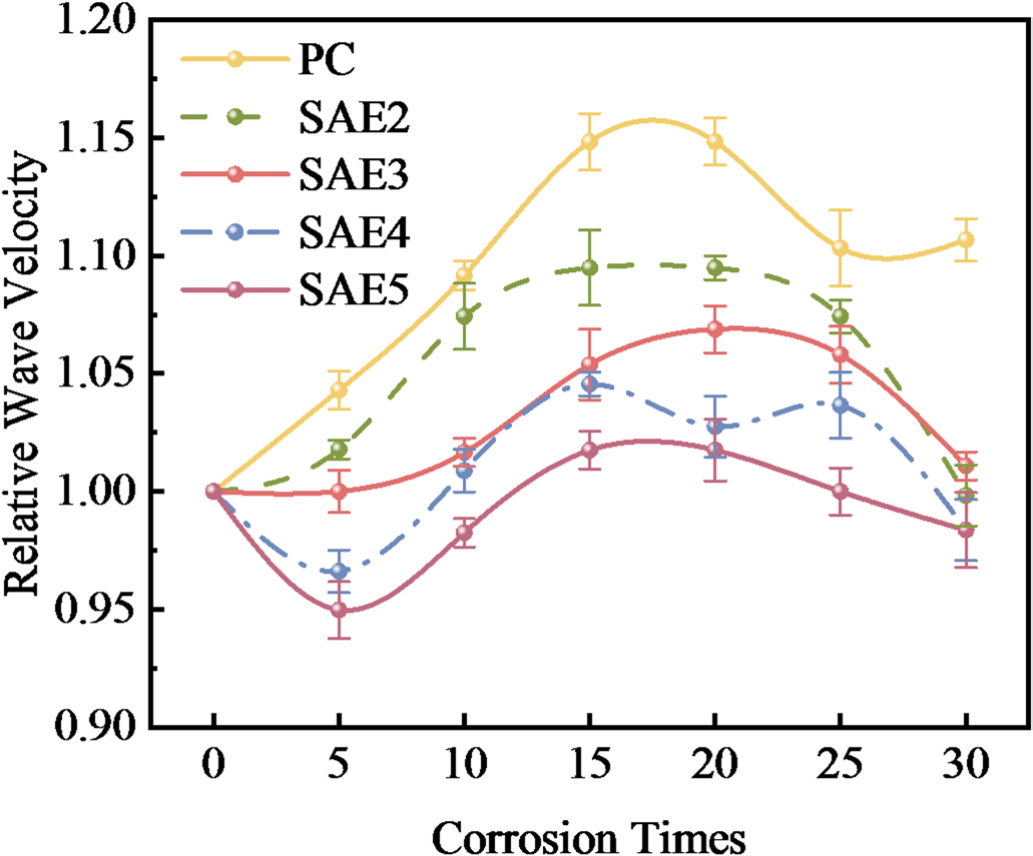
The variation of RWV of SAE/CaCO_3_ modified HSCC at different stages of sulfate erosion.

Ultrasonic testing, a non-destructive evaluation technique, enables rapid detection and quantification of internal defects through acoustic impedance discontinuity analysis. To minimize measurement variability, this study adopted relative wave velocity (RWV) as the damage evaluation parameter^27,30^. Figure 11 illustrates the RWV evolution of SAE/CaCO_3_ modified HSCC under sulfate attack. All specimens displayed an initial RWV increase followed by progressive decline. This indicates that in the early stage of sulfate attack, due to the formation of erosion products and the infiltration of sulfate, the modified HSCC matrix becomes more compact, thus the RWV of the sample shows an upward trend; as the erosion further expands, the sample is affected by expansion stress and sulfate dissolution, resulting in micro-cracks and defects, causing the RWV to start to decline.

A comparative analysis of the RWV in samples with varying SAE contents reveals that the variation amplitude of RWV significantly decreases with increasing SAE dosage. This phenomenon can be attributed to SAE’s pore-refining effect on the cement matrix microstructure, which effectively restricts sulfate penetration and subsequently inhibits the formation of expansive erosion products. During the initial sulfate attack phase, the PC specimens without SAE exhibit greater wave velocity variations due to their denser internal structure resulting from substantial sulfate ingress. However, it is anticipated that prolonged exposure will induce substantially higher internal damage accumulation in PC specimens compared to SAE-modified samples. This prediction aligns with the findings presented in Section “The regularity of mass variation”, demonstrating that SAE incorporation enhances the sulfate attack resistance of cement-based materials through microstructural optimization. Similarly, this verification aligns with the variation characteristics of compressive strength presented in Section “The changing regularity of flexural and compressive strength”. Specifically, the compressive strength of sample PC exhibits the most pronounced decrease, whereas the compressive strengths of specimens SAE4 and SAE5 demonstrate an increasing trend.

### Microscopic mechanism analysis

#### Analysis of XRD patterns of corrosion products

**Fig. 12:**
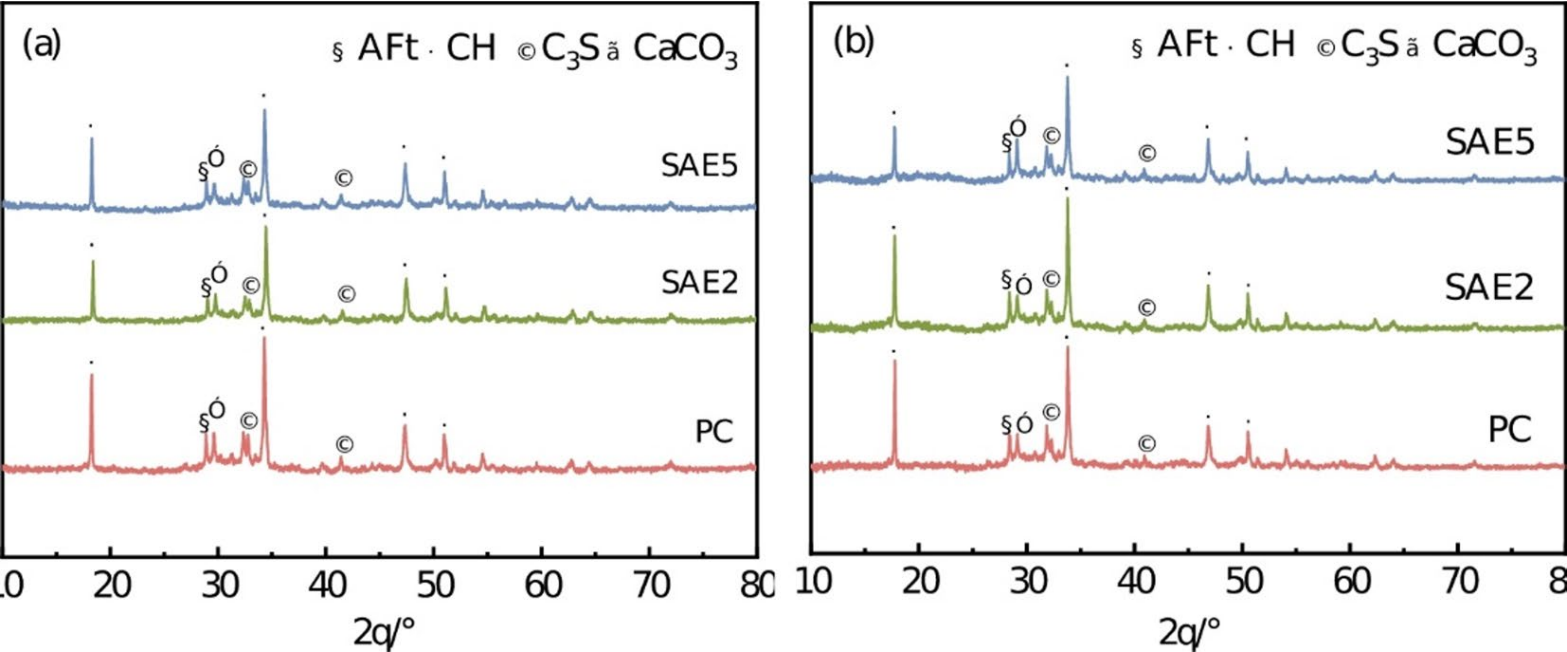
XRD patterns of SAE/CaCO_3_ modified HSCC at different sulfate attack stages: (**a**) C15; (**b**) C30.

Figure 12 presents a comparative analysis of XRD patterns for SAE/CaCO_3_ modified HSCC at different sulfate attack stages. Notably, specimens subjected to 30 wet-dry cycles exhibit enhanced diffraction peak intensities for CH, ettringite (C_6_AsH_32_, AFt), and CaCO_3_ compared to those exposed to 15 cycles. The amplification of CH and CaCO_3_ peaks demonstrates a dose-dependent relationship with SAE content.

Existing studies indicate that SO_4_^2−^ progressively infiltrate cementitious matrices during cyclic wet-dry exposures in Na_2_SO_4_ environments, reacting with hydration products to form crystalline corrosion products^31^. During sulfate attack, infiltrated SO_4_^2−^ initially reacts with CH to generate gypsum. Given the low activation energy of AFt formation, gypsum subsequently reacts with residual unhydrated C_3_A or aluminum-bearing phases (e.g., C_4_AH_13_ or C_4_AsH_12_) to produce secondary AFt, as described by the following reaction. This process persists until aluminum phases are fully consumed, after which gypsum accumulates as a stable byproduct^32,33^.

5 $$S{O_4}^{{2-}}+CH+2{H_2}O \to CaS{O_4} \cdot 2{H_2}O+2O{H^{-} }$$

6 $${C_3}A+3CaS{O_4} \cdot 2{H_2}O+26{H_2}O \to {C_6}As{H_{32}}$$

7 $${C_4}A{H_{13}}+3CaS{O_4} \cdot 2{H_2}O+14{H_2}O \to {C_6}As{H_{32}}+CH$$

8 $${C_4}As{H_{12}}+CaS{O_4} \cdot 2{H_2}O+16{H_2}O \to {C_6}As{H_{32}}+CH$$

The corrosion mechanism elucidates why AFt and CH contents increase significantly in Fig. 12. Concurrently, with the further development of sulfate erosion, the hydration inside the cement is still ongoing. Under the synergistic interaction of SAE and CaCO_3_, CH and CaCO_3_ contents progressively rise. CaCO_3_ acts as a physical filler, densifying the matrix and thereby inhibiting SO_4_^2−^ ingress. The microstructural synergism between SAE and CaCO_3_ will be thoroughly investigated in subsequent mechanistic analyses.

#### DSC analysis of corrosion products

**Fig. 13:**
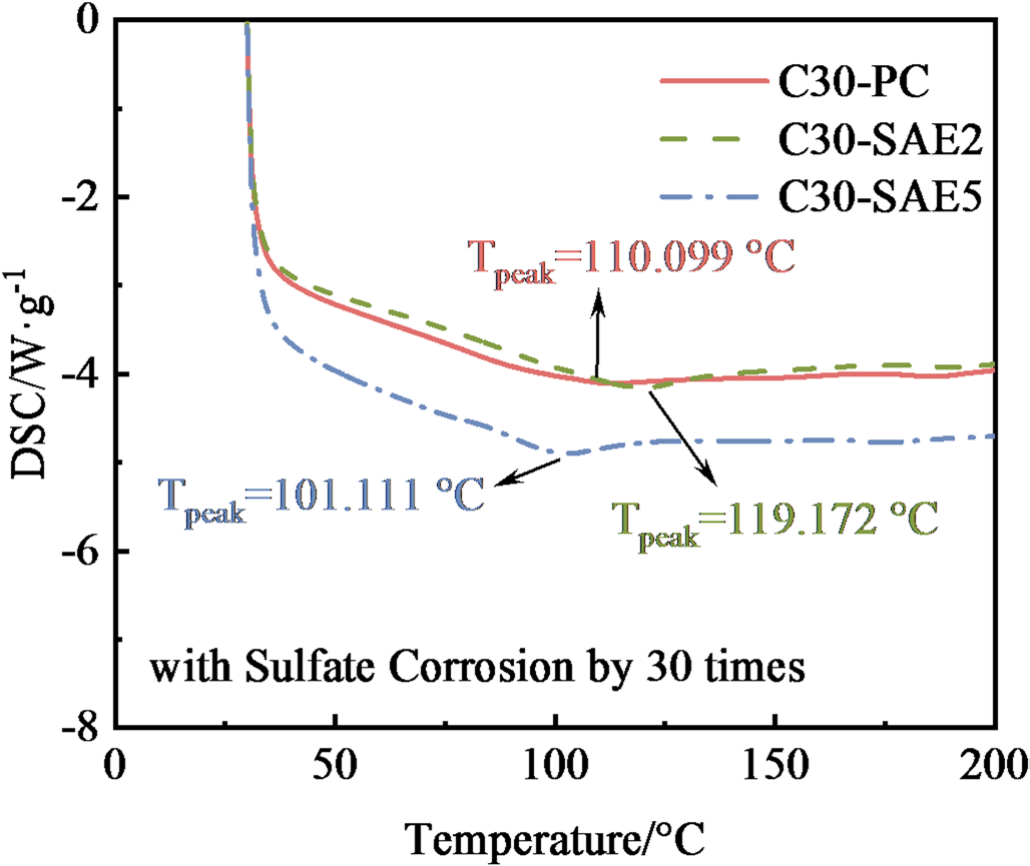
DSC curves of SAE/CaCO_3_ modified HSCC after 30 cycles of sulfate attack.

Differential Scanning Calorimetry (DSC) is a thermoanalytical technique that measures the difference in heat flux between a sample and a reference material under controlled temperature programming, enabling the investigation of phase transitions and thermal effects associated with chemical reactions^34^. By identifying the peak temperatures of endothermic events in DSC curves, characteristic reaction products can be determined. Table 5 summarizes the thermal signatures of key sulfate attack products in concrete. During Na_2_SO_4_ exposure, the primary corrosion products under wet conditions are typically AFt and gypsum, both exhibiting thermal effects below 200 °C (Table 5). Consequently, the analysis focuses on DSC peak features within this temperature range, as illustrated in Fig. 13.

**Table 5:** Thermal effect properties of related products during the sulfate attack on concrete^35–37^.

Peak temperature range (°C)	Thermal effect properties
80 –120	AFt dehydration decomposition
130 –170	Decomposition and dehydration of gypsum
About 450	CH decomposition
About 750	Decomposition of CaCO_3_

The DSC profiles reveal distinct endothermic peaks corresponding to AFt below 200 °C, while no gypsum decomposition signals are detected. This indicates that AFt dominates as the primary corrosion product in SAE/CaCO_3_ modified HSCC after 30 sulfate attack cycles. Prior studies establish that gypsum formation during sulfate attack requires either of two conditions:

1. Low Al_2_O_3_ content in cement, resulting in insufficient aluminum-bearing phases (e.g., C_3_A) to fully consume sulfate-generated gypsum (Eqs. 5, and 6);
2. High Al_2_O_3_ content coupled with elevated SO_4_^2−^ concentrations, which reduce pore solution pH, destabilizing AFt and triggering its retrograde decomposition into gypsum^34^.

In this study, the low-concentration sulfate solution and the cement’s high Al_2_O_3_ content (> 5 wt%) preclude gypsum formation. The abundant aluminum phases effectively consume sulfate ions via AFt generation until complete depletion, aligning with the absence of gypsum-related thermal signatures in the DSC data. Meanwhile, the synergistic effect of SAE and CaCO_3_ enables them to act as nucleation sites within the cement matrix, accelerating hydration and consuming CH to form a stable monocarboaluminate phase^38^. This process inhibits the formation of gypsum and mitigates internal structural damage caused by the expansion of corrosion products. The DSC analysis results are consistent with the macroscopic performance changes, explaining from the micro level the excellent sulfate resistance of SAE/CaCO_3_ modified HSCC.

#### Comparative analysis of the structural characteristics of matrix pores

**Fig. 14:**
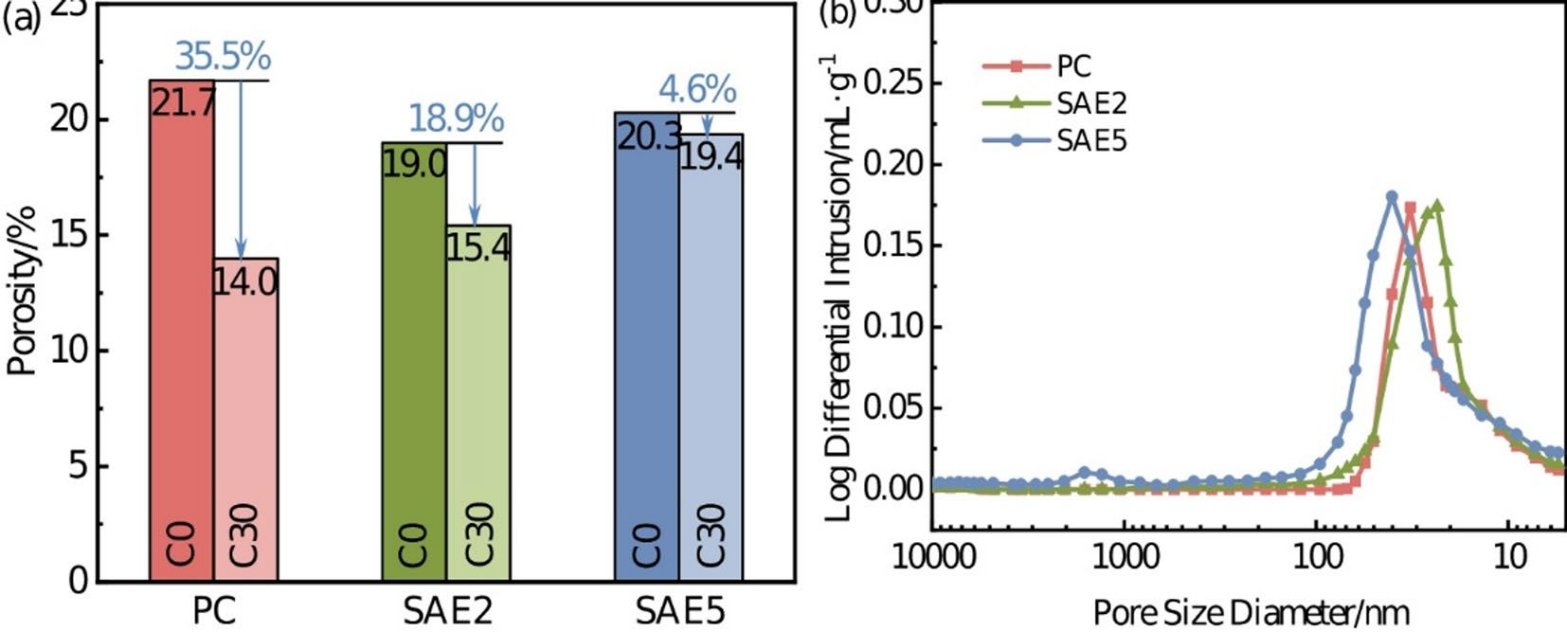
The pore structure change diagram of SAE/CaCO_3_ modified HSCC after 30 cycles of sulfate attack: (**a**) The change of total porosity before and after erosion; (**b**) The difference of mercury intrusion.

Figure 14 illustrates the pore structure evolution of SAE/CaCO_3_ modified HSCC after sulfate attack. Figure 14a quantifies total porosity variations before and after sulfate exposure, while Fig. 14b delineates mercury intrusion differentials across various pore size ranges. Pre-exposure data indicated that the porosity of the PC specimens was significantly higher than that of the SAE-modified specimens. Conversely, post-erosion analyses reveal an inverse correlation: total porosity escalates proportionally to SAE dosage.

Notably, sulfate exposure universally reduces total porosity across all specimens, albeit with diminishing efficacy at higher SAE concentrations. This phenomenon arises from sulfate-induced AFt crystallization (Eqs. 5–8), where corrosion products preferentially nucleate within pores and microcracks. Needle-shaped AFt fills internal voids, thereby decreasing overall porosity. Previous studies have established that increasing the SAE dosage substantially elevates the proportion of larger gel pores within the matrix^18^. Classified as harmless pores in cementitious systems, these gel pores hinder SO_4_^2−^ migration, thereby inhibiting the sulfate attack progression and enhancing the specimens’ sulfate resistance. This mechanism is corroborated by the lower rate of porosity change observed in specimens SAE5 (Fig. 14a) after sulfate exposure.

Critical pore size analysis in Fig. 14b corroborates these findings: post-erosion, PC and SAE2 specimens display denser matrices compared to SAE5. Paradoxically, this apparent pore refinement detrimentally impacts macroscopic performance. AFt crystallization generates internal expansive stresses, while its preferential growth at pore-microcrack interfaces weakens interfacial bonding and induces structural friability in the affected zones^34^. These microstructural degradations align with the mechanical strength deterioration of SAE/CaCO_3_ modified HSCC discussed in Section “The changing regularity of flexural and compressive strength”, confirming the dual role of AFt in pore occlusion and mechanical compromise.

#### Microscopic morphology feature analysis of corrosion products

**Fig. 15:**
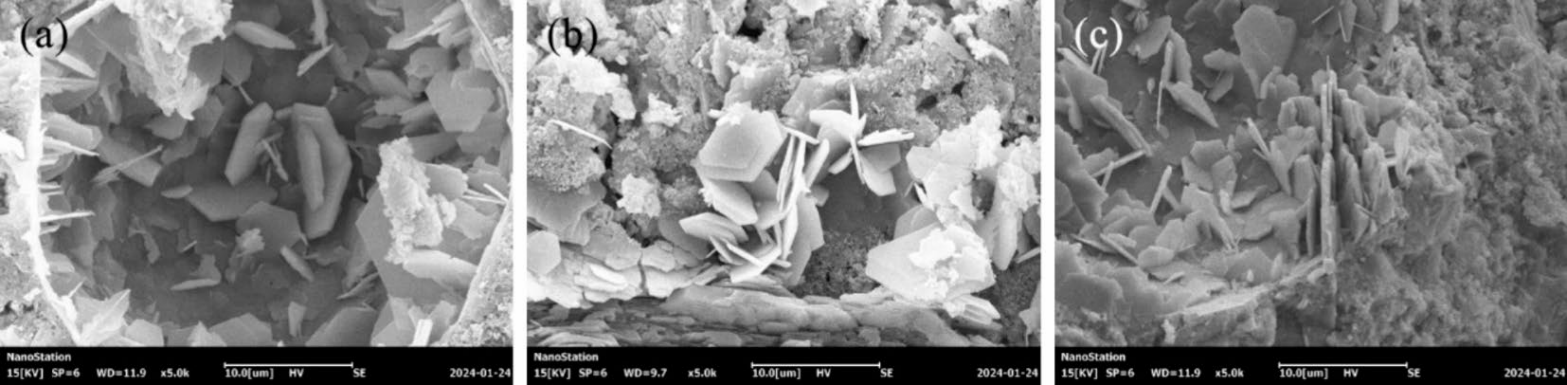
SEM images of of SAE/CaCO_3_ modified HSCC after 30 cycles of sulfate attack: (**a**) C30-PC; (**b**) C30-SAE2; (**c**) C30-SAE5.

Figure 15 depicts microstructural evolution of SAE/CaCO_3_ modified HSCC after sulfate attack, focusing on the morphological changes of AFt and CH within the pore structure. Notably, specimen PC exhibited significantly more acicular ettringite formation within the pores, consistent with XRD findings in Section “Analysis of XRD patterns of corrosion products”.

Furthermore, observation of the CH morphology changes in Fig. 15 reveals that as the SAE dosage increases, CH exhibits a stacked lamellar morphology. This is attributed to the substantial reduction in CH crystal accumulation caused by the increased SAE content^39^. Adsorbed polymers thermodynamically stabilize the primary nuclei of CH deposits by reducing the interfacial energy, thereby strongly inhibiting subsequent crystal growth^40^. Consequently, within the SAE/CaCO_3_ modified HSCC cement matrix, the crystal morphology of CH progressively manifests as stacked lamellae with increasing SAE dosage^18^.

In summary, sulfate attack on cementitious matrices manifests through dual pathways:

1. Physical effect: SO_4_^2−^ infiltrates interconnected capillary pores, where cyclic crystallization during wet-dry cycles generates internal stresses and microcracking, ultimately causing surface spalling.
2. Chemical effect: SO_4_^2−^ reacts with hydration products to form expansive phases (e.g., AFt, gypsum), while also accelerating decomposition of cementitious binders. These processes collectively reduce interfacial cohesion and mechanical integrity, culminating in structural failure^26,27,34,36^.

## Conclusions

This study investigates SAE/CaCO_3_ modified HSCC with SAE dosages of 0%, 2%, 3%, 4%, and 5% (by total binder mass), exploring the synergistic enhancement mechanisms of SAE and CaCO_3_ on sulfate resistance. Key conclusions are summarized as follows:

1. The sulfate resistance of SAE/CaCO_3_ modified HSCC improves with increasing SAE content. Macroscopically, specimens with higher SAE dosages exhibit lower mass variation rates and reduced mechanical performance degradation. For instance, after 30 sulfate wet-dry cycles, the compressive strength of SAE5 specimens reaches 82.6 MPa, representing a 7.55% enhancement compared to non-eroded counterparts of the same age.
2. Microstructural analyses reveal that the primary mechanism for improved sulfate resistance in SAE/CaCO_3_ modified HSCC originates from SAE-induced pore structure refinement. The SAE polymer forms a gel network that obstructs capillary pore connectivity, effectively limiting SO_4_^2−^ ingress and suppressing the formation of expansive AFt. Additionally, CaCO_3_ synergizes with SAE by acting as a reactive filler, densifying the matrix and further reducing porosity.
3. The study establishes a critical correlation between macroscopic properties and microstructural evolution. SAE/CaCO_3_ modification inhibits sulfate-induced microcracking and interfacial debonding by restricting SO_4_^2−^ penetration, which serves as a key factor in enhancing sulfate resistance. Notably, while SAE incorporation increases the F/C ratio, prolonged sulfate exposure gradually diminishes this advantage, indicating progressive interfacial degradation at the polymer-cement matrix interface.
4. The SAE/CaCO_3_ modification strategy provides a viable solution for improving the durability of cementitious materials in aggressive environments (e.g., coastal and underground engineering). Optimizing SAE dosage (e.g., 4–5%) achieves a balanced compromise between mechanical performance, sulfate resistance, and cost-effectiveness.

Further research should focus on long-term durability under coupled environmental stress factors, such as chloride-sulfate interactions. Furthermore, to facilitate the practical engineering application of this modification technology, incorporating supplementary cementitious materials (SCMs) like fly ash, silica fume, or other solid waste materials to alter the binder composition is essential. This approach is necessary not only to reduce costs but also to pursue more environmentally sustainable outcomes.

## Data Availability

All data generated or analyzed during this study are included in this published article.
